# A multi-omic integrative approach combining m^6^A-epitranscriptomic, transcriptomic, and splicing alternative events reveals potential candidates for colorectal cancer diagnosis

**DOI:** 10.1016/j.gendis.2025.101537

**Published:** 2025-01-22

**Authors:** Hatim Boughanem, Jesus Pilo, Alejandro Rego, Libia Ajendra Garcia-Flores, Teresa Dawid-de Vera, Francisco J. Tinahones, Gracia Maria Martin-Nuñez, Manuel Macias-González

**Affiliations:** a Unidad de Gestión Clínica de Endocrinología y Nutrición, Hospital Universitario Virgen de la Victoria, Malaga 29010, Spain; b Instituto de Investigación Biomédica de Málaga y Plataforma en Nanomedicina-IBIMA Plataforma BIONAND, Malaga 29010, Spain; c CIBER Fisiopatologia Obesidad y Nutricion (CIBEROBN), Instituto de Salud Carlos III, Madrid 28029, Spain; d Lipids and Atherosclerosis Unit, Department of Internal Medicine, Hospital Universitario Reina Sofía, Cordoba 14004, Spain; e Maimonides Institute for Biomedical Research in Cordoba (IMIBIC), Cordoba 14004, Spain; f Unidad de Gestión Clínica (UGC) de Anatomía Patológica, Instituto de Investigación Biomédica de Málaga (IBIMA), Hospital Universitario Virgen de la Victoria, Málaga 29010, Spain

Colorectal cancer (CRC) continues to be the third most frequently diagnosed cancer, and the second leading cause of cancer-related mortality. Several non-invasive biomarkers have emerged, but only a few have been incorporated into clinical practice due to the lack of sensitivity.^1^ Research on the epigenome has unveiled potential clinical applications for diagnosis and therapy response.^2^^,^^3^ Particularly, recent evidence suggests a novel role of RNA methylation in the development of CRC,^4^ revealing an overall RNA m^6^A hypomethylation.^5^ However, our understanding of their contribution to CRC remains limited. To address this, we investigated m^6^A modification in CRC using an integrative approach. High-throughput sequencing was performed to analyze the m^6^A-epitranscriptome (methylated RNA immunoprecipitation sequencing; m^6^A), transcriptome (mRNA), and alternative splicing events (AS; RNA sequencing) in leukocytes from both healthy participants (*n* = 16) and patients with CRC (*n* = 15) (Table S1 summarizes the baseline characteristics of the participants) from the “Virgen de la Victoria” University Hospital, Málaga, Spain.

As for the m^6^A analysis, the principal component analysis of total normalized m^6^A peak profiles revealed a partial separation, as observed in mRNA and AS analyses (Fig. S1A, B), and clustering of both groups, where PC1 explains 91% of the total variance, and PC2 accounts for 3% (Fig. 1A). In terms of normalized counts, patients with CRC showed decreased IP counts (fewer m^6^A modifications) when compared with controls (*p* < 0.001) (Fig. S1C). Opposite results were observed for AS (*p* < 0.001), but not significant for mRNA (*p* = 0.180) (Fig. S1D, E). The analysis revealed that the enrichment of m6A-modified peaks over the genome was highest in the 3′ untranslated region (UTR), followed by the coding DNA sequence (CDS) and 5′ UTRs (Fig. 1B; Fig. S1F). This hypomethylation was observed in 3′ UTR, 5′ UTR, and CDS regions when control and CRC patients were compared (all *p* < 0.001) (Fig. 1C). More than 75% of differential m^6^A peaks are located in the intron region. The remaining are located in the exon region, 3′ UTR, and 5′ UTR (Fig. 1D). The differential analysis of m^6^A resulted in a total of 113,062 peaks differentially methylated, in which 834 were hypermethylated and 112,228 peaks were hypomethylated (Table S2). On the other hand, the mRNA differential analysis (adjusted by age and sex) identified a total of 3857 significantly dysregulated genes (*p* < 0.05; 1194 up-regulated and 2663 down-regulated) (Table S2), while the differential analysis of AS revealed a total of 21,167 differentially AS events (*p* < 0.05; 14,213 AS events were increased and 6954 AS events were decreased) (Table S3).

**Figure 1:**
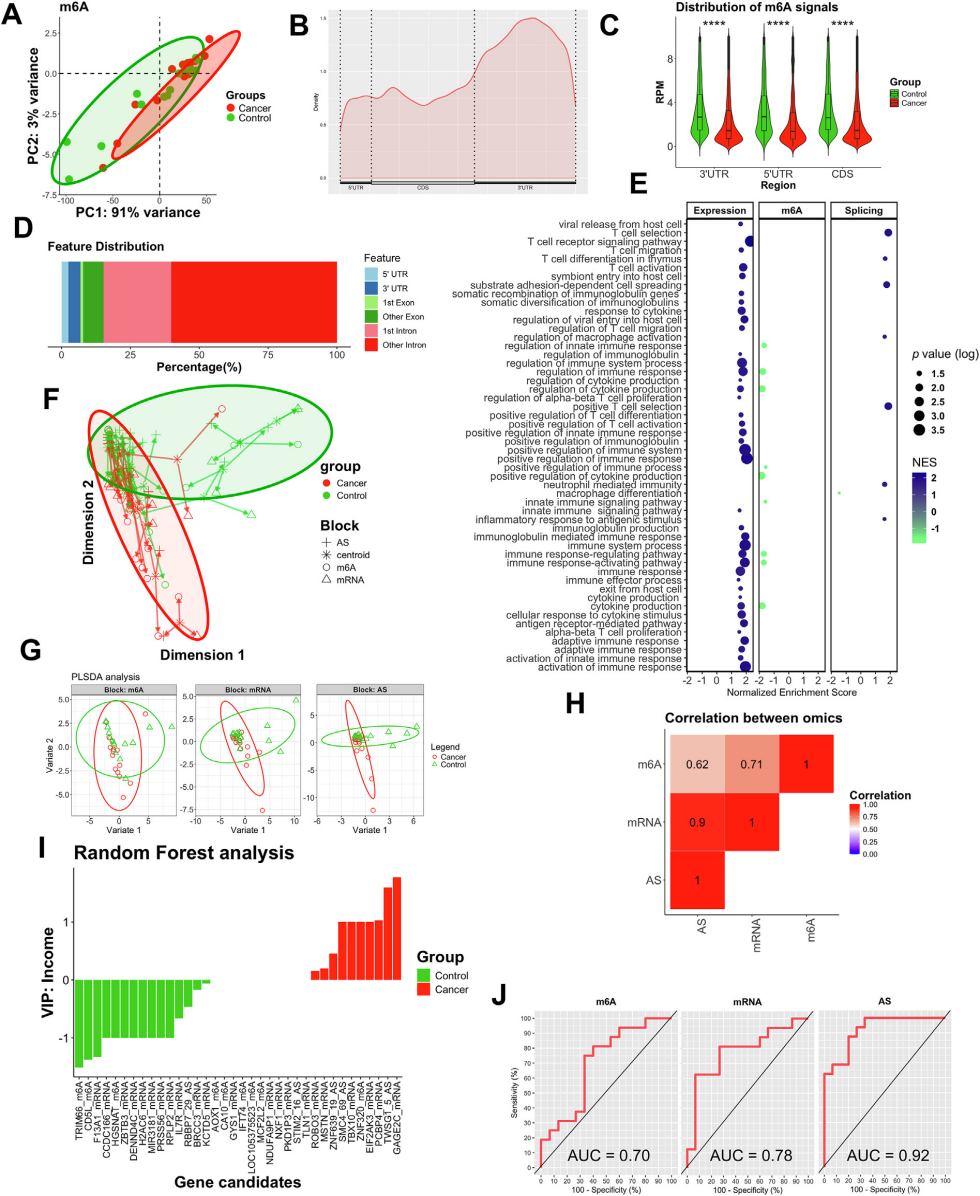
Multiomic integrative analysis combining m^6^A-epitranscriptomic, transcriptomic, and AS events in leukocytes in CRC. **(A)** PCA of m^6^A analysis plotting was conducted using transformed data on the log scale normalized to library size using the *DESeq2* package. A variance stabilizing transformation was applied to remove the dependence of variance on the mean, particularly addressing the high variance of the log counts when the mean is low. The percentage of global variation explained by each principal component is provided in the axis labels. **(B)** Metagene profile of m^6^A distribution across the transcriptome in controls and patients with CRC. **(C)** Normalized counts of m^6^A immunoprecipitated RNA, divided by 5′ UTR, 3′ UTR, and CDS regions. Asterisks indicate significant differences between the groups according to the Wilcoxon test (∗*p* < 0.05, ∗∗*p* < 0.01, ∗∗∗*p* < 0.001, ∗∗∗∗*p* < 0.0001). **(D)** The distribution of the m^6^A peaks along the transcript, including the 5′ UTR, 3′ UTR, first and other exons, as well as first and other introns. We used the *ChIPseeker* R package to annotate the genomic region of the peak. **(E)** Gene Set Enrichment Analysis was done using the following genes: For m^6^A, we applied an FDR <0.001 with an absolute LogFC greater than 2.5, and genes that had more than three m^6^A peaks (479 genes). For mRNA, we identified genes with an absolute LogFC greater than or equal to 1.2 and a *p*-value ≤0.01 (450 genes). For AS, we applied an absolute LogFC greater than 2 and a *p*-value <0.001 (291 genes). **(F)** Arrow plot from multiblock sPLS-DA performed on the data of integrated dataset of epitranscriptomic, transcriptomic, and AS events. The samples are projected into the space spanned by the first two components for each dataset and then overlaid across datasets. The start (tail) of the arrow indicates the location of the centroid of all datasets (blocks) for samples. The end (tip) of each arrow indicates the location of samples in each block, projected onto the averaged latent components. **(G)** sPLS-DA consensus plot for the combination of the three datasets showing complete discrimination of groups of datasets. **(H)** Sample scatterplot from plotDiablo displaying Pearson correlation between each component (lower diagonal plot). **(I)** Random forest analysis of the most important variables for predicting the presence and the absence of CRC. **(J)** ROC curve analysis of m^6^A, Mrna, and AS analysis for the prediction of CRC. AS, alternative splicing; CRC, colorectal cancer; FDR, false discovery rate; LogFC, log fold change; NES, normalized enrichment score; PC, principal component; PCA, principal component analysis; ROC, reciever operating curve; sPLS-DA, sparse partial least squares regression for discrimination analysis; TSS, transcription start site; TTS, transcription termination site; UTR, untranslated region.

To refine the selection of potential candidate and determine their biological functions, we applied several stringent filters. For m^6^A, we applied a false discovery rate lower than 0.001 with an absolute Log foldchange (LogFC) greater than 2.5, and genes with more than three m^6^A peaks (479 m^6^A peaks). For mRNA, we identified genes with an absolute LogFC greater than or equal to 1.2 and *p* ≤ 0.01 (450 genes). For AS, we applied an absolute LogFC greater than 2 and *p* < 0.001 (291 AS events). The gene set enrichment analysis (focused on the immune system) revealed an increase in immune response-related pathways when focusing on mRNA and AS, such as T cell regulation, cytokine production, and regulation of immune response. In contrast, m^6^A analysis showed a decrease in the innate immune response (Fig. 1E). The Gene Ontology (GO) and Kyoto Encyclopedia of Genes and Genomes (KEGG) analyses supported these findings. The functional role of the dysregulated m^6^A marks showed a dysregulation in metabolic function in CRC (Fig. S1G). The mRNA analysis showed processes related to immune system regulation and cytokine signaling (Fig. S1H). Finally, AS analysis showed processes related to metabolic dysregulation (Fig. S1I), suggesting that all three omics demonstrate dysregulation in both the immune system and the metabolic function of immune cells.

Finally, to further investigate the relationship between the three omics, we applied a holistic and unbiased multilevel analysis using multiblock analysis, which is a multivariate data dimensionality reduction method for the integration of multiple data. In Figure 1F, the start of the arrow indicates the centroid between all datasets for samples, and the tips of the arrows indicate the location of that sample in each block. Short arrows indicated a high level of agreement between the three datasets in the healthy group, while moderate agreement was observed between the three datasets in the CRC group. Overall, there were short arrows regarding the AS analysis, indicating high agreement. The combination of the three datasets provided clear clustering between groups. The first component of sparse partial least-squares discriminant analysis of the combined m^6^A-epitranscriptomic, transcriptomic, and AS datasets clearly discriminated healthy participants from patients with CRC, with the AS data showing the highest discriminatory capacity (Fig. 1G; Fig. S1J). Additionally, we observed strong correlations between the three datasets: m^6^A and mRNA (*r* = 0.71), between m^6^A and AS (*r* = 0.62), and between mRNA and AS (*r* = 0.90) (Fig. 1H), suggesting a high correlation between omics. Fig. S1K represents the multi-omics molecular signature for each sample. Blocks of homogeneous color depict subsets of features from each dataset (green: AS events; blue: m^6^A; red: mRNA). To potentially select candidate markers for CRC diagnosis, we selected candidates from the integrative analysis (Fig. S1L) and conducted a random forest analysis. This analysis clearly positioned candidates as the most important variable to predict the presence of CRC. Genes like *GAGE2C* (mRNA), *TWSGT* (AS), *PCBP4* (mRNA), ZNF320 (m^6^A), *TBX10* (mRNA), *SMC4* (AS), *MSTN* (mRNA), *ROBO3* (mRNA), and *TLN1* (mRNA) increased in CRC, while other genes such as *TRIM66* (m^6^A), CD5L (m^6^A), and *F13A1* (mRNA) increased in controls (Fig. 1I). This multi-omic analysis showed an area under the curve value of 0.70 for m^6^A, 0.78 for mRNA, and 0.92 for AS events, having the best predictive model (Fig. 1J).

In summary, we present novel findings regarding the m^6^A, mRNA, and AS events in CRC. Our data suggest that there is a global m^6^A hypomethylation, along with dysregulation of transcriptomic and AS events, affecting specific immune system status and metabolic functions of leukocytes. The integrative analysis clearly displayed a correlation between these three omics and identified specific genes highly associated with CRC, as the random forest showed. This offers a new perspective on the impact of RNA methylation on the development of CRC, but also other related mechanisms. The mechanistic function could probably be due to mechanisms related to systemic inflammation, as high-sensitivity C-reactive protein levels were increased in CRC patients (Table S1). Elevated systemic inflammation could dysregulate immune cells at both central and peripheral levels, promoting the production of specific immune cells that prime an inflammatory state. This, in turn, leads to significant changes in their metabolism and function, including changes in AS and their impact on gene expression. Here, we present a new epigenetic regulatory mechanism involving m^6^A. The overall hypomethylation may offer a new diagnostic approach but also indicates a dysregulation in the homeostasis of the epitranscriptome in the context of cancer. However, further research is needed to establish biological pathways implicated as well as mechanistic approaches for these dysregulations.

## Ethics declaration

The study was conducted in accordance with the guidelines laid down in the Declaration of Helsinki. This study was reviewed and approved by the Ethics and Research Committee of the University Hospital “Virgen de la Victoria” (Reference code: 0311/PI7). Written informed consent was obtained from all patients, and all clinical investigations were conducted according to the principles of the Declaration of Helsinki.

## Funding

This study was supported by the “Centro de Investigacion Biomédica en Red Fisiopatología de la Obesidad y Nutricion”, which is an initiative of the “Instituto de Salud Carlos III” (ISCIII) of Spain, financed by the European Regional Development Fund under “A way to make Europe"/"Investing in your future” (CB06/03), a grant from ISCIII (No. PI18/01399, PI21/00633); UMA-FEDERJA-085, from Programa Operativo FEDER 2014–2020 of the Consejería de Economía y Conocimiento de la Junta de Andalucía; and a grant from the Consejeria Universidad, Investigacion e Innovacion Junta de Andalucia (No. PY20- 01270, PI0293-2019). H.B. was supported by a predoctoral fellowship “Plan Propio IBIMA 2020 A.1 Contratos predoctorales” (No. predoc20_002) and by a “Sara Borrell” postdoctoral contract (No. CD22/00053) from the Instituto de Salud Carlos III—Madrid (Spain), “Financiado por la Unión Europea—NextGenerationEU”, and the plan Recuperación, Transformación y Resiliencia. L.A.G.-F. was supported by a “Sara Borrell” postdoctoral contract (No. CD21/000131) from the Instituto de Salud Carlos III—Madrid (Spain). G.M.M.-N. was supported by a postdoctoral contract from the University of Malaga (No. UMA20-FEDERJA-092). M.M.G. was the recipient of the Nicolas Monardes Programme from the “Servicio Andaluz de Salud, Junta de Andalucia”, Spain (No. RC-0001-2018, C-0029-2014).

## CRediT authorship contribution statement

**Hatim Boughanem:** Writing – review & editing, Writing – original draft, Software, Methodology, Investigation, Formal analysis, Data curation, Conceptualization. **Jesus Pilo:** Methodology, Investigation, Data curation. **Alejandro Rego:** Investigation, Conceptualization. **Libia Ajendra Garcia-Flores:** Methodology, Investigation. **Teresa Dawid-de Vera:** Methodology, Investigation. **Francisco J. Tinahones:** Writing – review & editing, Supervision, Project administration, Investigation. **Gracia Maria Martin-Nuñez:** Writing – review & editing, Methodology, Investigation, Funding acquisition, Data curation, Conceptualization. **Manuel Macias-González:** Writing – review & editing, Writing – original draft, Validation, Supervision, Investigation, Funding acquisition, Data curation, Conceptualization.

## Data availability

The data are available upon request from the corresponding authors.

## Conflict of interests

The authors declared no conflict of interests.

## Appendix A Supplementary data

Multimedia component 1 Multimedia component 1

Figure S1A detailed and continued multi-omic integrative analysis combining m^6^A-epitranscriptomic, transcriptomic, and AS events in leukocytes in CRC. **(A, B)** PCA plots for (A) mRNA and (B) AS analysis conducted using transformed data on the log scale normalized to library size with the *DESeq2* package. A variance stabilizing transformation was applied to remove the dependence of variance on the mean, particularly addressing the high variance of the log counts when the mean is low. The percentage of global variation explained by each principal component is provided in the axis labels. **(C)** Normalized counts of m^6^A (outliers have been eliminated to increase the clarity of the figure). **(D, E)** mRNA (D) and AS (E) in both healthy participants and patients with CRC are presented. Significant differences between the groups were confirmed according to the Wilcoxon test. **(F)** m^6^A peak locations over the whole genome. **(G**–**I)** Gene Ontology and KEGG Pathway analysis and functional analysis were conducted using the (G) m^6^A peaks profile, (H) mRNA, and (I) AS analyses. Analysis was done using the following genes: for m^6^A, we applied an FDR <0.001 with an absolute LogFC greater than 2.5, and for genes that had more than three m^6^A peaks (479 genes); for mRNA, we identified genes with an absolute LogFC greater than or equal to 1.2 and a *P*-value ≤0.01 (450 genes); for AS, we applied an absolute LogFC greater than 2 and a *P*-value <0.001 (291 genes). **(J)** Clustered image map (Euclidean distance, complete linkage) of the multi-omics signature based on the first component. Samples are represented in rows, with selected features on the first component in columns (circle: AS events; triangle: mRNA variables; square: m^6^A variables; red: patients with CRC; green: healthy participants). **(K)** Clustered image maps from the PLS were applied to multi-omic data (AS events, mRNA, and m^6^A variables) in patients with CRC and healthy participants. Colors indicate different omic layers and groups (light green: AS events; light red: mRNA variables; purple: m^6^A variables; red: cancer patients; green: healthy participants). **(L**) The most significant variables, ranked by the absolute value of their coefficients, are arranged from bottom to top. Since this is a supervised analysis, the colors correspond to the class with the highest median expression value for each feature. AS, alternative splicing; CDS, coding DNA sequence; CRC, colorectal cancer; FDR, false discovery rate; KEGG, Kyoto Encyclopedia of Genes and Genomes; LogFC, log fold change; mRNA, Transcriptomics; PC, principal component; PCA, principal component analysis; PLS, partial least squares. Figure
