# Supplementary material for: A multi-omic integrative approach combining m6A-epitranscriptomic, transcriptomic, and splicing alternative events reveals potential candidates for colorectal cancer diagnosis
Source: Genes Dis. 2025 Jan 22;12(6):101537. doi: 10.1016/j.gendis.2025.101537 (PMC12343466; doi:10.1016/j.gendis.2025.101537)
Supplement: Multimedia component 1 [file mmc1.docx]

**Materials and Methods**

*Study design and participants*

This investigation involved participants recruited from the University Hospital “Virgen de la Victoria” between 2012 and 2014. The study encompassed 16 patients diagnosed with CRC who underwent curative surgery. The diagnosis was confirmed by a pathology specialist through biopsy and colonoscopy, with all medical records and pathological examinations corroborating the findings. Biopsy samples were categorized by pathologists based on histological features and adhered to the "World Health Organization Classification of Tumors of the Digestive System" (2016). Patients with CRC underwent hemicolectomy and lower anterior resection with ileostomy (attributed to colorectal carcinoma), followed by total meso-colorectal excision. A minimum 5-year follow-up was conducted, involving clinical visits every three months for the initial two years and subsequently every six months from the third year onward. Each checkup encompassed the measurement of biochemical variables, a physical examination, biochemical tests, and a colonoscopy. Additionally, the study included 16 healthy participants (without CRC) who had undergone hiatus hernia surgery or cholecystectomy. Participants with inflammatory acute and chronic diseases, familial polyposis, or those patients who had chemotherapy or radiotherapy treatments were excluded. Participants with infection, renal or cardiovascular diseases, or patients who received treatment that altered their lipid and glucose profiles were also excluded. All participants gave written informed consent. The study was conducted in accordance with the guidelines laid down in the Declaration of Helsinki. This study was reviewed and approved by the Ethics and Research Committee of the University Hospital “Virgen de la Victoria” (Reference code: 0311/PI7).

*Samples and measurement included in the study*

Blood samples were collected from all participants. Plasma and buffy coat were obtained from blood samples collected in EDTA tubes, following centrifugation at 4,000 r.p.m. for 15 minutes at 4ºC. Serum samples were extracted from blood samples collected in tubes with separator gel through centrifugation at 4,000 r.p.m. for 15 minutes at 4ºC.

For RNA extraction, total RNA was isolated from the buffy coat using Total RNA Purification Kits (Norgen Biotek Corp., Canada) following the manufacturer's instructions. The quantity, purity, and quality of isolated RNA were assessed using a Nanodrop ND-1000 v3.5.2 spectrophotometer (Nanodrop Technology®, Cambridge, UK), ensuring an A260/280 ratio > 2. Additionally, evaluation was performed through electrophoresis gel, and the fragment size distribution of RNA fragments was examined using the Agilent 2100 Bioanalyzer (Agilent Technologies, USA) with the Agilent RNA 6000 Nano Kit (Agilent Technologies, USA). Samples with fragments below 200 base pairs were excluded.

*Laboratory measurements*

We utilized the Dimension Autoanalyzer (Dade Behring Inc., Deerfield, IL, USA) to assess levels of fasting glucose, total cholesterol, triglycerides, and high-density lipoprotein (HDL) cholesterol. Low-density lipoprotein (LDL) cholesterol was determined using the Friedewald equation. Fasting insulin levels were quantified through radioimmunoassay methods by BioSource International Inc. (Camarillo, CA, USA). Insulin resistance homeostasis model assessment (HOMA-IR) was computed using the formula: HOMA-IR = fasting insulin (IU/mL) × fasting glucose (mmol/L)/22.5. Carcinoembryonic Antigen (CEA) and carbohydrate antigen 19.9 (CA19.9) were assessed via ELISA (DRG diagnostics, Germany).

*m^6^A RNA Immunoprecipitation and RNA Sequencing*

For RNA fragmentation, 2-10 μg of RNA was fragmented by incubating the samples at 70°C in fragmentation buffer (100 mM Tris-HCl, 100 mM ZnCl_2_). Total fragmentation time per sample was determined by their RNA degradation profile (0-3 min). The fragment size distribution of the RNA fragments was checked using the Agilent 2100 Bioanalyzer (Agilent Technologies, USA) with the Agilent RNA 6000 Nano Kit (Agilent Technologies, USA). Subsequently, m^6^A enrichment was performed. The sample was divided into two aliquots: one for the m^6^A-immunoprecipitation procedure (IP sample) and the other to be used as a non-immunoprecipitated input control (INput sample). The IP RNA aliquot was incubated for 2 h at 4°C with an antibody-bead mixture, containing the following: anti-m^6^A antibody (Monoclonal mouse purified IgG, m6A - 202 111, SySy Antibody against N6-methyladenosine modifications of RNA and DNA), protein-A and protein-G magnetic beads (10002D and 10004D, respectively, Thermo Fisher Scientific), and RNasin Plus RNase Inhibitor (N2611, Promega, Madison, WI). After extensive washing, the m^6^A-enriched RNA was purified using the RNeasy MiniElute spin column (QIAGEN). The RNA quality is assessed using the On-Chip Electrophoresis system with the Bioanalyzer 2100 equipment (Agilent Technologies, USA) to determine the RIN (amount and size of isolated RNA). A concentration of ≥ 20 ng/μl of INput RNA and IP-RNA eluted from m^6^A-IP was utilized for library preparation and RNA sequencing. For library preparation, the SMARTer Stranded Total RNA-Seq Kit v2 - Pico Input Mammalian was used strictly following the manufacturer’s instructions. Samples were dual-indexed to allow post-sequencing demultiplexing. The fragment size distribution of the libraries were checked in the Agilent 2100 Bioanalyzer (using the Agilent HS DNA Kit). The libraries were purified using the Mag-Bind RXNPure Plus magnetic beads (Omega Biotek), following the instructions provided by the manufacturer, and quantified with the Qubit dsDNA HS Assay Kit (Thermo Fisher Scientific). Then, they were pooled in equimolar amounts according to the Qubit results. The resulting pool was sequenced in a fraction of a NovaSeq PE150 flow cell, aiming for a total output of 10 gigabases per sample.

*Bioinformatic analysis*

The raw FASTQ files undergo quality checks using FastQC v0.11.9 (Andrews, 2010) and were summarized with MultiQC v.1.13.dev0 (Ewels et al., 2016). Trimmomatic 0.39 (Bolger et al., 2014) was utilized to eliminate adapters (ILLUMINACLIP option) and low-quality regions (AVGQUAL:26, TRAILING:25, and HEADCROP:10). Reads shorter than 75 base pairs (bp) were discarded from the dataset. Before mapping, SortMeRNA (Kopylova et al., 2012) was employed to identify rRNA reads using the Homo sapiens rRNA 45S pre-ribosomal N5 (NCBI reference sequence NR_046235.3) as the reference sequence. The filtered reads were aligned to the Homo sapiens hg38 genome reference using STAR 2.7.8a (Dobin et al., 2013). Secondary alignments were removed, and only uniquely aligned reads with a mapping quality greater than 30 (mapQ>=30) were retained for further analysis. The resulting BAM mapping files and their corresponding index files were used for subsequent analysis steps.

The differential modification analysis was conducted using Rstudio and the Bioconductor packages ExomePeak2 and ChIPSeaker, which facilitate the detection of RNA modification peaks and differential modification sites. For RNA-Seq analysis, we evaluated genes that exhibited statistically significant differential expression between healthy and patients with CRC. This analysis was conducted using the Bioconductor package DESeq2, designed for the R statistical programming environment after adjusting by age and gender. We employed DEXSeq, a Bioconductor package tailored for detecting differential exon usage based on RNA-seq data, to model the read counts of all exons under different conditions, utilizing a negative binomial distribution. Significance testing for an interaction term between exon and condition was conducted (FDR-adjusted P-value < 0.05).

For summarizing and visualizing the m^6^A methylome data, we executed principal component analysis (PCA) on the immunoprecipitated read counts. These counts were adjusted for expression level variation to reduce data dimensionality. The first two principal components were displayed in a PCA plot. In order to perform inferential tests, we modeled the preprocessed immunoprecipitation read counts. Finally, employing an FDR (false discovery rate) cut-off of 0.05, we merged all connected significant bins and reported the genomic locations of differential m^6^A peaks. Pathway and gene ontology enrichment analysis are performed using G:Profiler with default settings.

*Statistical analysis*

The variables are presented as mean ± standard deviation (SD) for continuous variables and as numbers (percentages) for categorical variables. Depending on the normality of the variables, a Student t-test or Mann-Whitney test was applied. All analyses and graphical representations were conducted using R v.3.5.1 software (Integrated Development for R, RStudio, PBC, Boston, MA, USA), and the significance threshold was set at *p* < 0.05.
