# Supplementary figures and images for: A multi-omic integrative approach combining m6A-epitranscriptomic, transcriptomic, and splicing alternative events reveals potential candidates for colorectal cancer diagnosis
Source: Genes Dis. 2025 Jan 22;12(6):101537. doi: 10.1016/j.gendis.2025.101537 (PMC12343466; doi:10.1016/j.gendis.2025.101537)

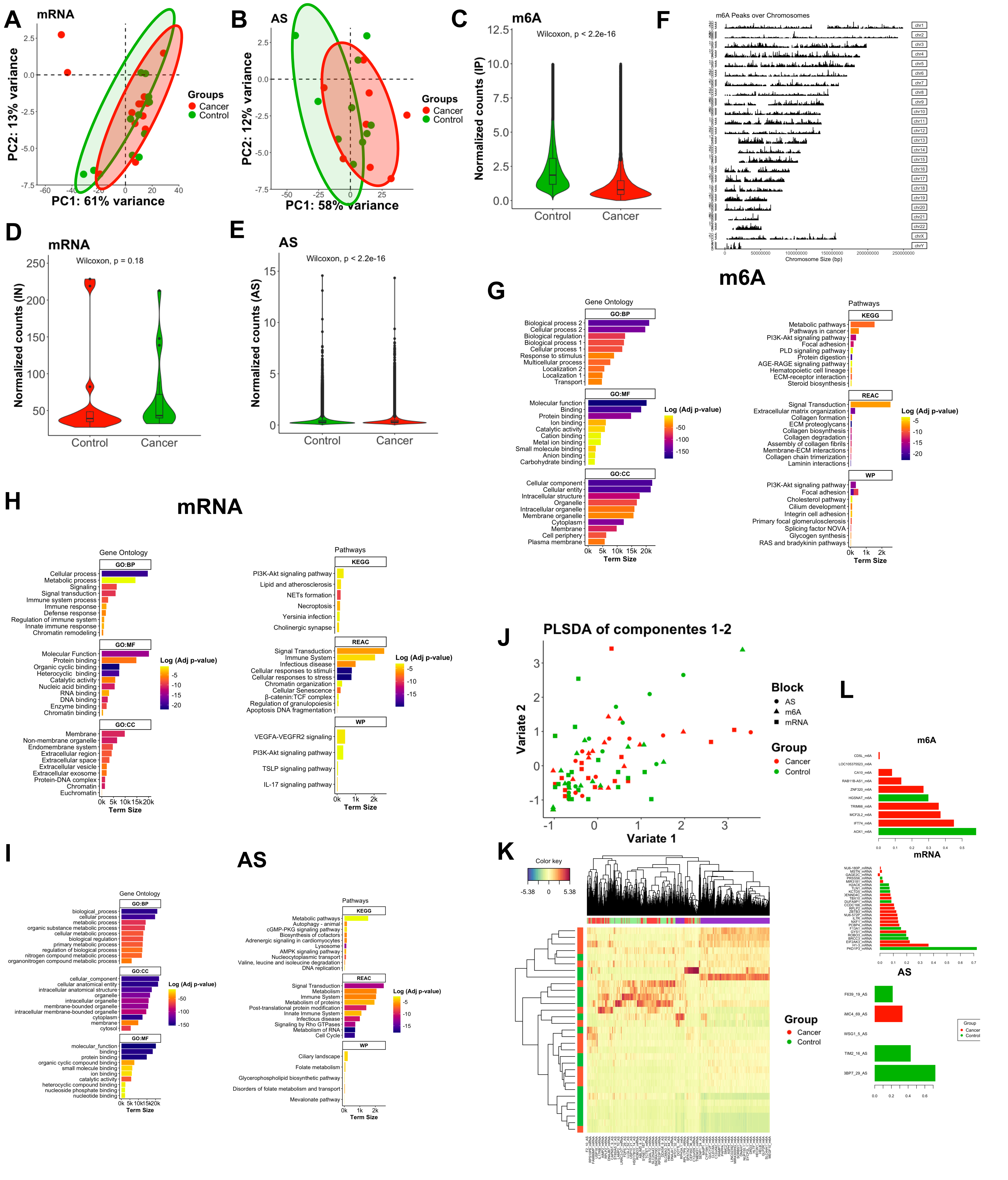

Supplement: Figure — S1A detailed and continued multi-omic integrative analysis combining m6A-epitranscriptomic, transcriptomic, and AS events in leukocytes in CRC. (A, B) PCA plots for (A) mRNA and (B) AS analysis conducted using transformed data on the log scale normalized to library size with the DESeq2 package. A variance stabilizing transformation was applied to remove the dependence of variance on the mean, particularly addressing the high variance of the log counts when the mean is low. The percentage of global variation explained by each principal component is provided in the axis labels. (C) Normalized counts of m6A (outliers have been eliminated to increase the clarity of the figure). (D, E) mRNA (D) and AS (E) in both healthy participants and patients with CRC are presented. Significant differences between the groups were confirmed according to the Wilcoxon test. (F) m6A peak locations over the whole genome. (G–I) Gene Ontology and KEGG Pathway analysis and functional analysis were conducted using the (G) m6A peaks profile, (H) mRNA, and (I) AS analyses. Analysis was done using the following genes: for m6A, we applied an FDR <0.001 with an absolute LogFC greater than 2.5, and for genes that had more than three m6A peaks (479 genes); for mRNA, we identified genes with an absolute LogFC greater than or equal to 1.2 and a P-value ≤0.01 (450 genes); for AS, we applied an absolute LogFC greater than 2 and a P-value <0.001 (291 genes). (J) Clustered image map (Euclidean distance, complete linkage) of the multi-omics signature based on the first component. Samples are represented in rows, with selected features on the first component in columns (circle: AS events; triangle: mRNA variables; square: m6A variables; red: patients with CRC; green: healthy participants). (K) Clustered image maps from the PLS were applied to multi-omic data (AS events, mRNA, and m6A variables) in patients with CRC and healthy participants. Colors indicate different omic layers and groups (light green: AS e [file mmc2.pdf]
