# Supplementary material for: A multi-omic integrative approach combining m6A-epitranscriptomic, transcriptomic, and splicing alternative events reveals potential candidates for colorectal cancer diagnosis
Source: Genes Dis. 2025 Jan 22;12(6):101537. doi: 10.1016/j.gendis.2025.101537 (PMC12343466; doi:10.1016/j.gendis.2025.101537)
Supplement: Multimedia component 3 [file mmc3.docx]

**Table 1.** Baseline characteristics of the participants included in the study

| **Variables** | **All participants** | **Control** | **Patients with CRC** | ***p* value** |
| --- | --- | --- | --- | --- |
|  | ***N=31*** | ***N=16*** | ***N=15*** |  |
| Age, years | 60.8 (11.8) | 55.3 (11.2) | 66.7 (9.70) | 0.005** |
| Sex, |  |  |  | 1.000 |
| Males, | 17 (54.8%) | 9 (56.2%) | 8 (53.3%) |  |
| Females, | 14 (45.2%) | 7 (43.8%) | 7 (46.7%) |  |
| BMI, kg/m^2^ | 27.8 (6.96) | 28.5 (8.77) | 27.0 (4.51) | 0.563 |
| Glucose, mg/dL | 99.5 (22.5) | 91.2 (20.2) | 108 (22.1) | 0.034* |
| Insulin, mg/dL | 7.47 (4.49) | 9.56 (4.01) | 5.37 (4.04) | 0.008** |
| HbA1c, % | 5.65 (0.43) | 5.61 (0.28) | 5.69 (0.57) | 0.669 |
| HOMA-IR | 197 (43.4) | 209 (42.1) | 185 (42.5) | 0.124 |
| Total cholesterol, mg/dL | 141 (62.1) | 117 (55.6) | 166 (60.2) | 0.026* |
| Triglycerides, mg/dL | 123 (28.1) | 133 (25.5) | 113 (28.0) | 0.049* |
| LDL, mg/dL | 47.0 (16.6) | 52.0 (17.3) | 41.7 (14.6) | 0.084 |
| HDL, mg/dL | 1.73 (1.18) | 2.03 (1.13) | 1.47 (1.20) | 0.215 |
| hsCRP, mg/dL | 12.3 (17.8) | 3.94 (2.15) | 16.7 (20.9) | 0.034* |

Data are represented as mean (SD) or n (%). Groups were divided according to the presence or absence of colorectal cancer. Asterisk indicates significant difference between groups according to the Mann Whitney test and Chi squared test was used for variables expressed as percentage (****p*<0.001, ***p*<0.01, **p*<0.05). **Abbreviations**. BMI: Body mass index, HbA1c: Hemoglobin glycosylated; HDL: high density lipoprotein; HOMA-IR, Homeostasis model assessment-insulin resistance. hsCRP: High-sensitive C reactive protein;
